# A high-throughput cell migration assay using scratch wound healing, a comparison of image-based readout methods

**DOI:** 10.1186/1472-6750-4-21

**Published:** 2004-09-09

**Authors:** Justin C Yarrow, Zachary E Perlman, Nicholas J Westwood, Timothy J Mitchison

**Affiliations:** 1 Department of Systems Biology, Harvard Medical School, Boston, MA 02115, USA; 2 The Institute of Chemistry and Cell Biology (ICCB), Harvard Medical School, Boston, MA 02115, USA; 3 School of Chemistry and Centre for Biomolecular Sciences, University of St Andrews, North Haugh, St Andrews, UK

## Abstract

**Background:**

Cell migration is a complex phenomenon that requires the coordination of numerous cellular processes. Investigation of cell migration and its underlying biology is of interest to basic scientists and those in search of therapeutics. Current migration assays for screening small molecules, siRNAs, or other perturbations are difficult to perform in parallel at the scale required to screen large libraries.

**Results:**

We have adapted the commonly used scratch wound healing assay of tissue-culture cell monolayers to a 384 well plate format. By mechanically scratching the cell substrate with a pin array, we are able to create characteristically sized wounds in all wells of a 384 well plate. Imaging of the healing wounds with an automated fluorescence microscope allows us to distinguish perturbations that affect cell migration, morphology, and division. Readout requires ~1 hr per plate but is high in information content i.e. high content. We compare readouts using different imaging technologies, automated microscopy, scanners and a fluorescence macroscope, and evaluate the trade-off between information content and data acquisition rate.

**Conclusions:**

The adaptation of a wound healing assay to a 384 well format facilitates the study of aspects of cell migration, tissue reorganization, cell division, and other processes that underlie wound healing. This assay allows greater than 10,000 perturbations to be screened per day with a quantitative, high-content readout, and can also be used to characterize small numbers of perturbations in detail.

## Background

When wounded or scratched, cell monolayers respond to the disruption of cell-cell contacts and an increased concentration of growth factors at the wound margin by healing the wound through a combination of proliferation and migration [1-3]; these processes reflect the behavior of individual cells as well as the properties of the cell sheet as a surrogate tissue. To perform a wound healing assay, a wound is typically introduced in a cell monolayer using an object such as a pipette tip or syringe needle and the assay is performed on an individual coverslip or in a multiwell plate. The monolayers recover and heal the wound in a process that can be observed over a timecourse of 3–24 hrs. The wound heals in a stereotyped fashion – cells polarize toward the wound, initiate protrusion, migrate, and close the wound. Progression of these events can be monitored by manually imaging samples fixed at timepoints or by time-lapse microscopy.

Wound healing assays are a classic and commonly used method for studying cell migration and the biology underlying it [4]. They have been used with multiple cell types and, as the monolayers heal the wound in a characteristic manner, they have been used to study cell polarization, matrix remodeling, cell migration, and numerous other processes [5-7]. Wound healing assays have been used both for detailed cell biological studies and for the discovery and validation of small molecule leads and other perturbations that affect cell migration [8-11]. The role of the Rho family GTPases, Rac, Rho, and Cdc42, in the establishment of polarity and the regulation of actin cytoskeletal structures has been studied using wound healing [12-14], as has the role of p53 in migration [15], and orientation of the microtubule organization center (MTOC) and the Golgi apparatus [16-18]. The assay has also been used as a proxy for angiogenesis, metastasis, and other physiological and pathophysiological processes [19-24].

In order to perform high throughput screening of cell migration, we developed a wound healing assay in a 384 well plate format that does not require expensive reagents, provides consistently shaped wounds, can provide detailed information on numerous processes involved in cell migration, and provides a quantitative, information-rich readout. We use multiple imaging technologies to assay the results and compare their relative merits.

## Results

### Adaptation of wound healing to a 384 well format

**Figure 1:**
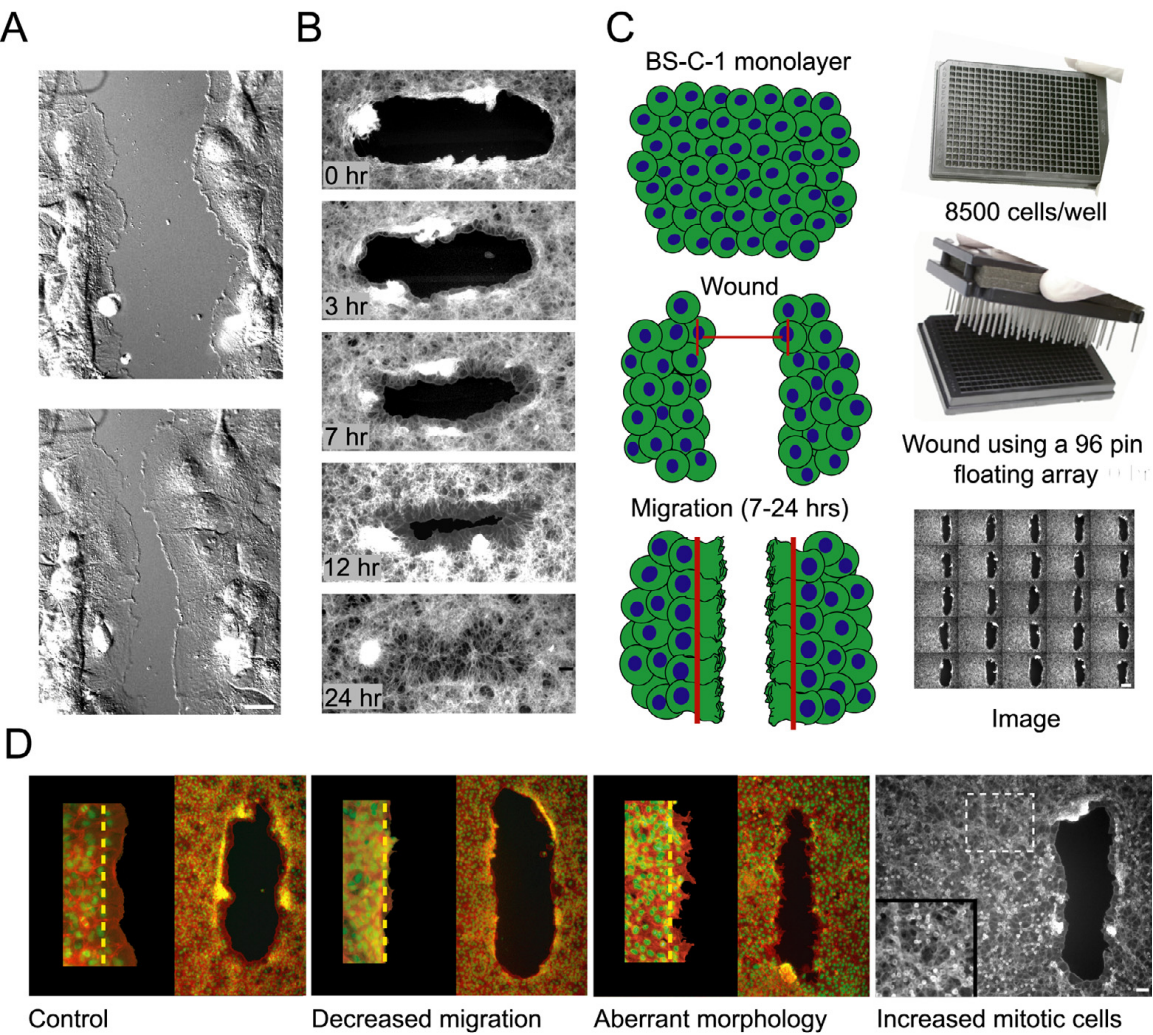
**Adaptation of a wound healing assay to a 384 well plate format. **(A) Images from a time-lapse sequence of BS-C-1 cells migrating on a coverslip to heal a wound. Bar 20 μm. (B) Images of wounds 0, 3, 7, 12, and 24 hrs after wounding show characteristic protrusion of lamella at 3 hrs, migration by 7 hrs, and eventual healing of the wound at 24 hrs. Cells are stained for filamentous actin. Bar 100 μm. (C) Schematic of the protocol used for screening. The 96 well pin array (shown) produces wounds with consistent shape and placement within each well. Image of 25 wells stained for filamentous actin 7 hrs after wounding. Bar 500 μm. (D) The primary phenotypes observed with an automated microscope in a small molecule screen (4× objective, 7 hr healing). Wells often showed a combination of these phenotypes. Cells are stained for filamentous actin and DNA. The dotted yellow line notes the approximate edge of the initial wound (based on the high density of nuclei). Bar 100 μm.

For the development of a high-throughput wound healing assay we chose to use BS-C-1 cells, a cell type with a classic wound healing response on glass coverslips [Figure 1A]. BS-C-1 cells were seeded in clear-bottom 384 well plates at high density and allowed to form monolayers overnight. We found that wound healing was observable between 3 and 24 hrs after wounding with a pipette tip or syringe needle. Significant cell migration could be seen at 3 hrs with lamella and protrusions at the wound margin. After 7 hrs, cell migration could be observed easily with a low magnification (4×) objective, and after 24 hrs wounds were completely healed [Figure 1B, Additional file: 1]. Because of the ease of distinguishing phenotypes at 7 and 24 hrs, we have used these timepoints for the assay.

To adapt this assay for parallel screening, we needed a method for introducing uniformly sized wounds in the same position of each well. We used a 96 well floating-pin transfer device – a tool primarily used for the transfer of solutions between plates. A floating pin array, with foam padding placed between the top plate and the pins, provides an adaptive stop to pin height and overcomes problems with plate planarity. We have adapted a 24-channel aspirator in the same manner for small-scale work.

To wound all 384 wells in a plate, the 96 well pin array is placed in the corner of a well, pushed down to engage all pins with the surface of the plate, and then moved laterally to produce the wound. This is then repeated in three neighboring wells to cover the plate and produce uniform wounds throughout [Figure 1C]. After the cells were wounded, we introduced perturbations to individual wells (in our case, small molecules). We then allowed the cells to recover for 7 hrs or 24 hrs before fixing, staining, and imaging each plate.

### Comparison of imaging technologies

We used four different imaging technologies to analyze the results of this assay, each with distinct advantages and drawbacks [summarized in Table 1]. We will discuss these approaches in order of image resolution, from highest to lowest: automated fluorescence microscopy after 7 hrs recovery, fluorescence and transmitted-light scanners after 7 hrs, and a fluorescence macroscope after 24 hrs.

### Automated microscopy

For highest resolution imaging of the assay we chose an end point of 7 hrs, when migration can be clearly seen, and used an automated fluorescence microscope to image individual wells after fixing and staining for filamentous actin and DNA [Figure 1D]. The microscope is a standard inverted fluorescence instrument. Augmented with an x-y stage, it moves between plate wells and a piezoelectric z-motor on the objective gives a focused image. Capturing images with a 4× objective provided sufficient resolution to determine the extent of migration and the morphology of the cells at the wound margin. From these data, we defined four distinct phenotypes [Figure 1D]. A control well shows polarization of the cells toward the wound and concerted migration of the cell sheet, with neighboring cells connected and moving together into the wound. Wells showing decreased migration or aberrant morphology are readily apparent by visual inspection as are wells showing an increase in the number of mitotic cells, which manifest as bright spheres (in the actin channel) in an otherwise intact and adherent monolayer [Figure 1D]. Phenotypes that cause disruption of the monolayer, are considered toxic though we have not shown them to be.

Using automated microscopy, the image resolution is relatively high and the time required to image an entire plate is relatively long. Imaging one 384-well plate takes ~1 hr at 4× magnification. At 10× magnification, ~1.5 hrs are required per plate because at higher magnification two images per well must be taken to ensure that the wound edge is captured. We found it more informative to observe the entirety of the wound at 4× rather than parts of it at 10×, despite the higher resolution in the latter case.

### Scanners

**Figure 2:**
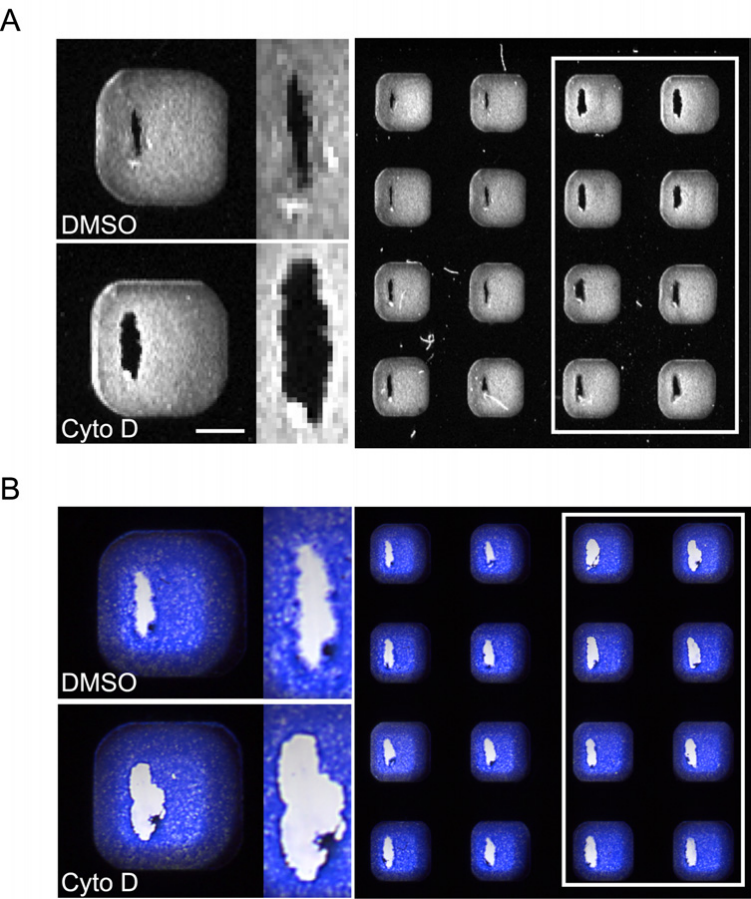
**The use of scanners to assay wound healing after 7 hrs recovery. **(A) An image of actin-stained wells obtained using a fluorescence scanner. Normal healing wounds (left two columns) can be distinguished from wells treated with a titration of the actin cytoskeleton disrupter cytochalasin D (white box) by the width of the wound and staining at the wound margin. (B) An image of Coomassie-stained wells obtained using a transmitted-light scanner. Normal healing wounds have a more diffuse wound margin, denoting cell migration into the wound, than wells treated with cytochalasin D (white box). Bar 1 mm.

A fluorescence scanner can be used to determine the extent of cell migration at the 7 hr timepoint. By staining filamentous actin and using a fluorescence scanner with a 42 μm/pixel resolution setting, we can observe consistent differences between normal and inhibited migration. Control wells show a veil of less-densely stained, migrating cells that extend into the wound with a concomitant decrease in wound width [Figure 2A]. Titration of a compound that blocks cell migration (the actin inhibitor cytochalasin D) shows complete inhibition of migration at 1 μM, as seen by a sharply delineated wound edge and a wider wound width [Figure 2A].

A simple transmitted-light scanner, normally used for scanning documents and costing less than $1,200, can also be used to monitor wound healing at 7 hrs and only requires that cells are stained with a dye. Figure 2B shows images of wells stained with Coomassie Brilliant Blue. Wells treated with cytochalasin D show inhibition of wound healing similar to that observed with the fluorescence scanner. Inhibited wells stain darkly at the wound margin while normal migration can be seen by a more diffuse wound margin, denoting migrating cells [Figure 2B].

In both cases, scanners do not provide specific information on cell morphology or other subtle effects in the 7 hr assay; however, acquisition time is greatly decreased. For the fluorescence scanner, acquisition time is 26 minutes at the resolution and image quality shown (42 μm/pixel and medium quality). For the conventional scanner, acquisition time is 8.5 minutes (at 10 μm/pixel, 2400 dpi) and cell staining with Coomassie Brilliant Blue takes only 10 minutes.

### Macroscope – Tundra or LeadSeeker

**Figure 3:**
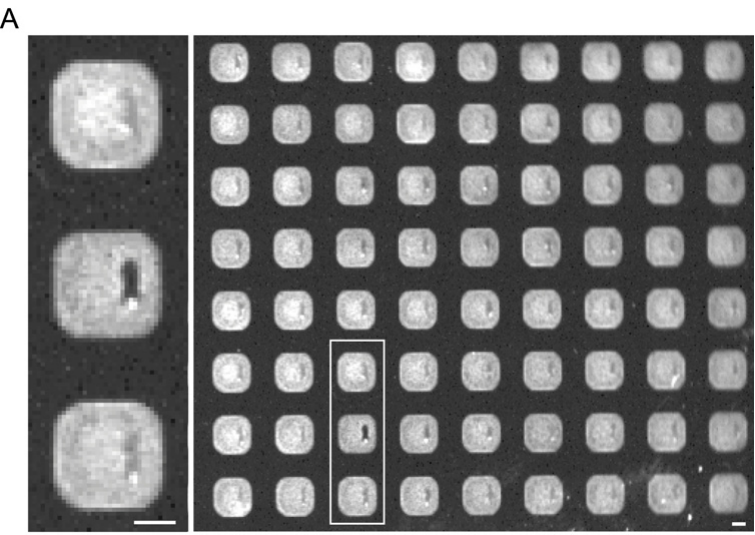
**The use of a fluorescence macroscope to assay wound healing after 24 hrs recovery. **Fluorescence macroscope image of wells stained for filamentous actin 24 hrs after wounding. A well showing reduced wound healing can be easily distinguished (middle well white box) from wells showing complete healing which are seen as light grey streaks. Bar 1 mm.

The lowest resolution imaging technology that we tested, a fluorescence macroscope had a resolution of ~100 μm/pixel. Detecting wound healing at this resolution required an incubation time of 24 hours after wounding as differences are not readily seen with this method at the 7 hr timepoint. Cells are wounded and allowed to recover for 24 hours before fixing, staining for filamentous actin, and imaging. The macroscope captures an image of the entire plate. At this magnification and resolution, an unhealed wound is seen as a non-staining, black streak within the monolayer [Figure 3]. In contrast, wounds that have healed completely are seen as lower-intensity, grey streaks.

The time required for acquisition of images using this technique, is limited only by the fluorescence signal and was typically ~5 seconds per plate.

### Automated image analysis of wound healing images

**Figure 4:**
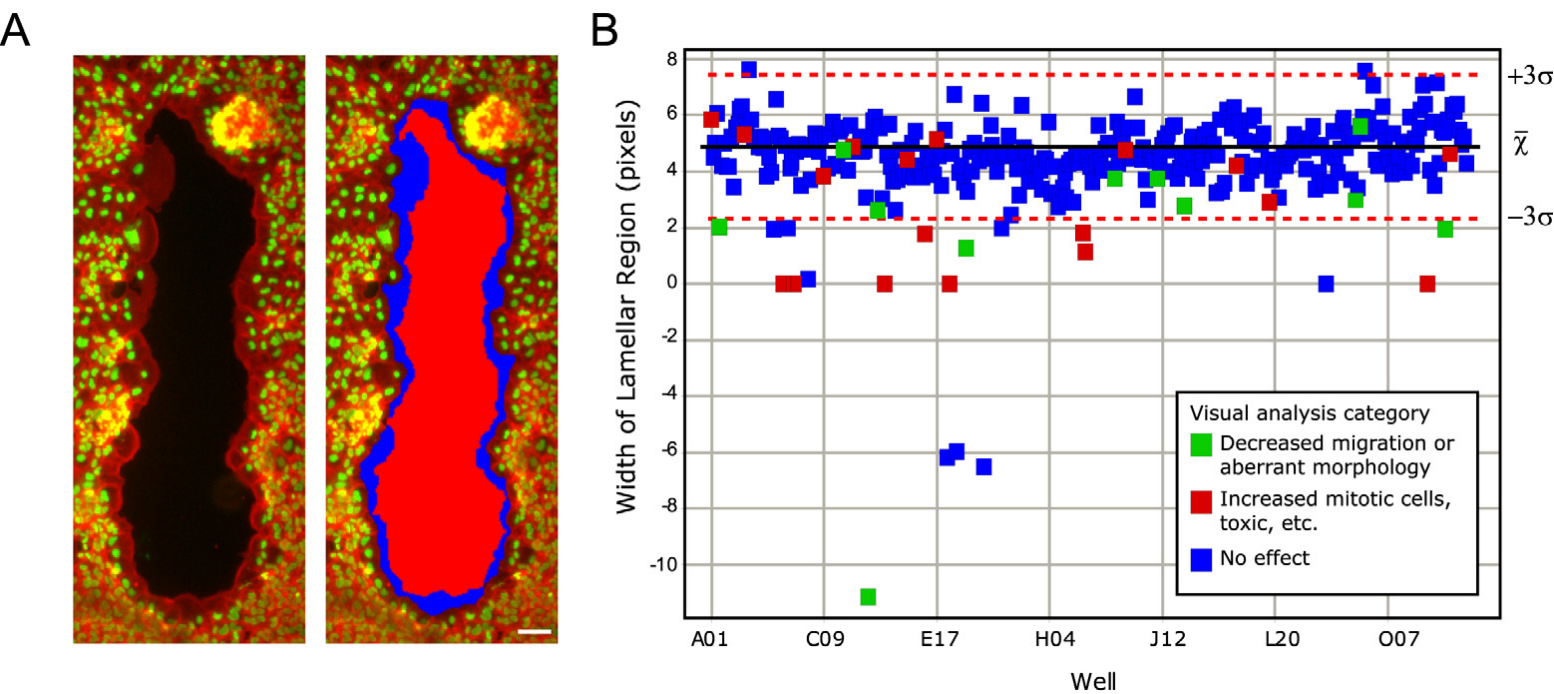
**Automated analysis of wound healing images. **(A) An image showing normal migration and the same image after automated analysis defines regions. Extracting characteristics of the lamellar region (blue) allows us to distinguish between the phenotypes that affect wound healing (Figure 1). Stains: red – filamentous actin; green – DNA. Bar 100 μm. (B) Automated and visual analysis are complementary approaches. Automatically generated lamellar width data is plotted for a 384 well plate. Compounds found to affect wound healing by visual analysis are shown in green, and wells that differed from the control in other ways (increased mitotics, a disrupted monolayer, etc.) are shown in red. Mean lamellar width (black line) and three standard deviations (red dashed lines) are plotted for control wells. Data collected from screening one 384 well plate of small molecules.

With all of the imaging techniques discussed here, we initially scored the assay by visual inspection. Visual inspection is fast, information-rich, and can distinguish subtle effects. This method proved useful during adaptation and optimization of the assay in high-throughput format. However, screening large numbers of perturbations by visual inspection is limited by subjectivity, operator fatigue, and the lack of quantifiable metrics. Thus, we developed an automated image analysis routine to provide a rapid and quantitative measurement of the wound healing assay with images captured using the automated microscope (automated methods for the other readouts could also be developed). Using images captured by automated microscopy with a 4× objective, we are able delineate measurable characteristics of the wound. After 7 hrs recovery, actin staining defines the extent of healing as well as the morphology of the cells and, because of cell polarization, DNA staining defines the approximate starting point of the migrating cells [Figure 4A]. Applying a standard set of image processing filters to threshold the image, we defined the area of the wound not staining for nuclei and the area not staining for actin. The difference between these two areas defines an annulus containing the lamella of the cells at the wound front (hereafter, lamellar region) [Figure 4A]. With the lamellar region defined, we can extract several metrics that characterize migration. Measurements that have been useful in quantifying the phenotypes of migration inhibition and aberrant morphology include: area of lamellar region, width of lamellar region, and the smoothness of the wound margin.

Automated analysis and visual inspection are complementary approaches. To illustrate this, we compare the two approaches using the results from one 384 well plate of a screen for small molecules that affect wound healing [Figure 4B]. The automated analysis values for each well are plotted and the average lamellar width from control wells is marked as a black line, with three standard deviations above and below that average marked as red dashed lines. Visual inspection of this same plate is illustrated on the graph by color-coding each well. Wells where migration is inhibited or morphology affected are shown in green; compounds that affect the well in other ways – wells with fewer cells, increased mitotic index, or disrupted monolayers – are shown in red; and compounds that show no significant deviation from control are shown in blue. As can be seen, the automated analysis picks up 4 wells that show inhibition of wound healing with lamellar widths more than 3 standard deviations from the mean and with less stringent bounds (2 standard deviations) 7 compounds are found to inhibit. Other compounds identified by visual inspection were not picked up by automated analysis.

### Validation of wound healing as a high-throughput assay

We have used methods described in this paper (notably, automated microscopy) to screen ~1,000 bioactive and ~20,000 random drug-like compounds. We were able to reproducibly identify compounds that affected wound healing with different effects including: inhibiting migration, affecting morphology, blocking completion of mitosis, and disrupting the cell monolayer. The details of these results will be presented elsewhere.

## Discussion

We present a cell migration assay in a 384 well plate format through the adaptation of tissue culture cell wound healing. We have also compared the readouts provided by four distinct imaging techniques technologies – automated microscopy, fluorescence and transmitted-light scanners, and a fluorescence macroscope – for their relative acquisition speed, image resolution, and information content.

High-content image-based screening is often performed at high magnification, however, we have found that low magnification images are information-rich and can be sufficient for observation of detailed phenomena. New screens utilizing imaging technologies are often explicitly developed to image plates at the highest resolution possible given time constraints (10× or higher), rather than at the lowest resolution required to discern differences between wells by visual inspection or automated analysis. Increased magnification further requires acquisition of a greater number of images in order to guard against sampling error. The more sophisticated imaging technologies also carry a hefty price tag. We show here that images taken at magnifications of 4× and lower still allow us to easily discern wells in which wound healing is inhibited and morphology affected. Low magnification imaging is an underutilized method and may be particularly effective for screens that monitor gross changes in protein localization such as nuclear transport, or protein transport from Golgi to plasma membrane; processes in which changes in localization might manifest as changes in the image texture of the stained cell monolayer.

While the choice of imaging technologies represents a tradeoff between resolution and information, we think that they should be used together rather than suggesting that they are mutually exclusive. One can iteratively image a given assay plate and take advantage of the best aspects of each technique. For example, a screen for inhibitors of cell migration could initially be performed using a lower resolution technique, taking advantage of the speed to identify interesting wells before imaging just those wells at higher resolution. Related to this, one can re-stain assay plates with different molecular markers. Plates initially screened with a marker that most easily defines the phenotype of interest can be subsequently re-stained with markers that provide different information. In this way, one can consider an assay plate a resource to which one can return to ask new questions.

Similar to the tradeoffs seen with different imaging technologies, different image processing techniques like automated image analysis and visual inspection have their distinct advantages but also provide complementary approaches. Our scheme for automated analysis of wound healing allows us to rapidly and easily identify wells in which wound healing is significantly inhibited or morphology clearly affected. However, the automated analysis missed a number of interesting wells that were identified by visual inspection. In part this is because the analysis is based on only a few measures – lamellar width, area, and smoothness. Any automated analysis based on measurable parameters will be limited to phenotypes that manifest along those parameters and might miss more subtle or complex phenotypes that "jump off the screen" when viewed by eye. Automated analysis can also be confounded by artifacts within the plate, dust particles, precipitates, or fluorescent small molecules. Instead of being thought of as a complete answer, automated analysis can enrich for wells that differ from the norm. We don't address automated analysis with the other imaging techniques, but from what can be seen by eye, automated approaches that measure the intensity of staining at the wound edge, or the steepness to which the intensity at the wound edge drops off should provide a good measure of wound healing at very low magnification and resolution.

As with any complex phenotypic assay, cell migration during wound healing can be inhibited by effects on global cellular processes – e.g. inhibition of protein translation, disruption of metabolism, disruption of ion homeostasis, etc. – and validation of the specificity of new perturbations is required. Even if the molecular target of the perturbation is unknown, this assay can be adapted or used to distinguish compounds that specifically inhibit or potentiate cell migration. For example, partial inhibition of migration with low concentrations of cytochalasin D could create a sensitized screen for cell migration and used to find suppressors or enhancers. Other complementary approaches include comparative screens across multiple cell lines that migrate differently and the use of specific trophic factors and cognate cell lines such as VEGF and endothelial cells. In our studies, we used still another approach: a series of counter screens that eliminated from our pool of hits, toxic compounds and compounds that do not inhibit processes of interest (Yarrow et. al. unpublished results). This approach has worked well and results will be reported elsewhere.

## Conclusions

The adaptation of a wound healing assay to a 384 well format facilitates the study of aspects of cell migration, tissue reorganization, cell division, and other processes that underlie wound healing. This assay allows greater than 10,000 perturbations to be screened per day with a quantitative, information-rich readout, and can also be used to characterize small numbers of perturbations in detail.

## Methods

### Tissue culture and 384 plate preparation

BS-C-1 (ATCC CCL-26) cells were grown in DMEM, 10% FCS, and antibiotics. Cells were plated in black 384 well plates with clear bottoms (Corning Costar 3712) at a density of 8500 cells/well in a volume of 50 μl using a liquid dispenser (Labsystems Multidrop). Plates were spun briefly at 500 rpm for ~30 s to in a tabletop centrifuge (Sorval RT7 plus). Cells were incubated overnight (37°C 5% CO_2_) and wounding was preformed 12 hours later.

### Wound healing assay, cell fixation, and staining

Wound healing was performed using a 96 well floating-pin transfer device with a pin diameter of 1.58 mm coming to a flat point at the tip with a diameter of 0.4 mm (VP Scientific VP-408FH). Foam backing was inserted between the plates of the pin array to provide a resistive stop and the external guide pins were bent to allow greater movement in the z-axis. The pin array was placed in the top corner of a well, pushed down into the plate to engage all pins, and then pulled toward the user. This was repeated in the three neighboring wells to cover all 384. Plates were returned to the tissue culture incubator for 7 or 24 hours before fixation.

Cells were fixed after removal of the media with a wand aspirator (VP scientific VP-186L) used along with the Labsystems Multidrop for all liquid handling. Fixation solution (100 mM K-Pipes pH 6.8, 10 mM EGTA, 1 mM MgCl_2_, 0.2% Triton X-100, 3.7% Formaldehyde) was added as 30 μl and incubated for 15 min. Wells were aspirated and washed 2× with TBS with 0.1% Triton-X 100 (TBS-Tx) and stained.

For experiments involving the automated microscope and macroscope, cells were stained in TBS-Tx with TRITC-phalloidin (Sigma P1591) 0.5 μg/ml and Hoechst (Sigma B2261) 0.1 μg/ml as 15 μl per well for 15 minutes. Wells were washed 2× with TBS-Tx and imaged. For experiments using the fluorescence plate scanner, after fixation, cells were incubated in TBS-Tx with 2% BSA (AbDil) for 30 minutes, incubated with mouse anti-actin antibody (Chemicon MAB1501) at 1:10,000 in AbDil for 45 minutes, washed 2× with TBS-Tx, incubated with secondary antibodies appropriate for the plate scanner (Molecular Probes A-21057), washed 2× with TBS-Tx and imaged. For experiments using the transmitted-light scanner, after fixation, cells were incubated with SDS-Page gel staining solution (0.25% Coomassie Brilliant Blue R-250, 50% methanol, 10% acetic acid) for 10 minutes, washed 2× with TBS and imaged.

### Imaging

#### Automated microscopy

We used a NikonTE300 inverted fluorescence microscope with filter wheel (Sutter Lamda10-2), x-y stage (Prior H107N300), and piezoelectric-motorized objective holder (Physik Instrumente P-723.10). Images were captured on a CCD camera (Hammamatsu OrcaER). Metamorph software (Universal Imaging Corporation) running the "Screen Acquisition" drop-in allowed coordination of software-based auto-focusing, movement between wells, imaging, and image evaluation. Images were acquired using a 4× or 10× objective with 2 × 2 binning. Exposure times were ~300 ms for TRITC-phalloidin and ~10 ms for Hoechst. An individual plate took ~1 hr to image at 4× and ~1.5 hrs at 10×. Individual black and white actin images (.tif) were compiled as a .stk file and scrolled through using keystrokes to visually annotate. Visual inspection of 1 plate of images (384) took 10 minutes.

#### Transmitted light scanner

An Epson 1680 scanner was used with the positive film setting. A scanning resolution of 1200 dpi (equivalent to 20 μm/pixel) gave an acceptable image with a read time for one plate of 3.5 minute. The image shown in Figure 2 was 2400 dpi (10 μm/pixel) and read time was 8.5 minutes. We also found that using the document scanning mode (with no transmitted light attachment) worked well. In this case, a lamp with a paper diffuser (placed on top of the plate) was used for even illumination.

#### Fluorescence scanner

An Odyssey scanner (LiCor) was tested at all combinations of scanning resolution and image quality. Scanning at a resolution setting of 42 um with medium image quality was found to be the optimal balance between speed and image quality. The scanner was used as per the manual, with no modifications.

#### Fluorescence macroscope

A Tundra macroscope, (Imaging Research – now available as the Leadseeker from Amersham [25]) a 12 cm telecentric lens with a N/A of 0.45, mounted with a -50°C cooled, thinned, back illuminated CCD camera for image capture, and a motorized stage to hold the plates – all enclosed in a light-tight box. The software was used as per the manual. To image the underside of plates, they were sealed (Corning Costar 6570) while containing TBS and inverted. Exposure times were ~5 seconds.

### Automated image analysis

Wound healing images were analyzed using software written using Visual Basic 6.0 (Microsoft) and Halcon 6.0.1 (MVTec Software) but could be implemented with most basic image analysis software. This software iterates the analysis over images specified by the Metamorph .HTS file and returns values to an Excel spreadsheet.

#### Non-DNA staining region (Additional file: 2A)

Hoechst images are convolved with a Laplacian-of-a-Gaussian (σ = 2 pixels) kernel. The resulting image is thresholded for pixels of value 0 and binarized. After a binary morphological opening (an erosion followed by a dilation) with a disc of radius 2.5 pixels, the largest contiguous region that does not touch the image edge is defined, and holes within this region are filled.

#### Non-actin staining region (Additional file: 2B)

A Kirsch edge detection filter is applied to actin images and the resulting image is thresholded at a manually set value (changed when needed to account for variation in staining intensity). The largest contiguous region that does not touch the image edge is defined and holes within this binary region are filled.

#### Definition of measures (in pixels)

*lamellar area: *area of annulus defined by the difference between the non-actin staining area and the non-DNA staining area; *lamellar width: *(lamellar area)/(perimeter length of the non-DNA staining region); *lamellar smoothness *(Additional file: 2C): (Perimeter of the non-actin staining region)/(perimeter of the morphological closing (dilation followed by erosion) of the non-actin staining region with a disc of radius 10 pixels).

## Authors' contributions

JCY developed the assay and analysis. ZEP wrote the software for automated analysis. NJW initiated a project for *in vivo *actin cytoskeleton small molecule inhibitors. TJM provided support and enthusiasm for the project.

## Supplementary Material

Additional file 1 **Wounds generated with the 96 well floating-pin array heal in a characteristic and measurable manner. **(A) Wounds generated with the 96 well floating-pin array healed for 0, 3, 7, 12, or 24 hours, were processed as above, and wound regions were measured using automated analysis. Normalized average values and standard deviations of both the area and median width of the non-actin staining region and the non-DNA staining region (see Methods for definitions) are shown. The non-DNA staining region at the 24 hr timepoint was automatically not measured (asterisks) because the non-actin staining region was 0. At least 24 wells of each condition were measured. Click here for file

Additional file 2 **Sample images illustrating automated analysis of wound healing images. **(A) Processing of DAPI image to generate non-DNA staining region. (B) Processing of actin image to generate non-actin staining image. (C) Comparison of lamellar wound region with its morphological closing allows assessment of lamellar smoothness. Click here for file

## Figures and Tables

**Table 1:** Comparison of four imaging methods to readout the wound healing assay

***Device***	***Resolution***	***Observable phenotypes***	***Timepoint for assaying***	***Acquisition speed***	***Cost of device***
Automated microscope	3 μm/pixel	Decreased wound healing, aberrant morphology, increased mitotic index	7 and 24 hr	~1 hr/plate with 4× ~1.5 hr/plate with 10×	~$200,000
Fluorescence scanner	42 μm/pixel	Decreased wound healing	7 and 24 hr	26 minutes/plate	~$50,000
Conventional scanner	10 μm/pixel	Decreased wound healing	7 and 24 hr	8.5 minutes/plate	~$1,000
Fluorescence macroscope	~100 μm/pixel	Decreased wound healing	24 hr	5 seconds/plate	~$500,000
