# Supplementary figures and images for: A high-throughput cell migration assay using scratch wound healing, a comparison of image-based readout methods
Source: BMC Biotechnol. 2004 Sep 9;4:21. doi: 10.1186/1472-6750-4-21 (PMC521074; doi:10.1186/1472-6750-4-21)

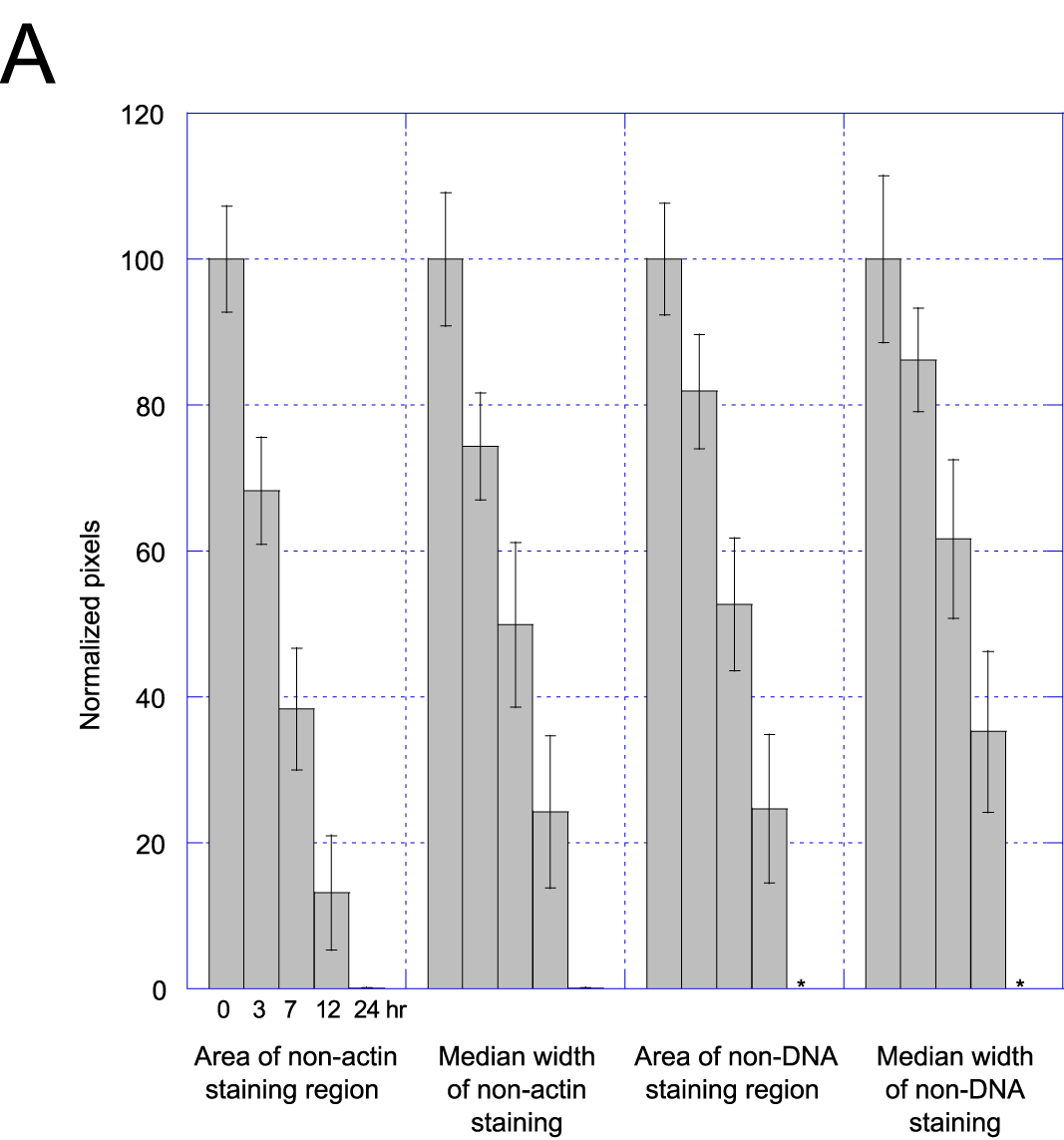

Supplement: Additional file 1 — Wounds generated with the 96 well floating-pin array heal in a characteristic and measurable manner. (A) Wounds generated with the 96 well floating-pin array healed for 0, 3, 7, 12, or 24 hours, were processed as above, and wound regions were measured using automated analysis. Normalized average values and standard deviations of both the area and median width of the non-actin staining region and the non-DNA staining region (see Methods for definitions) are shown. The non-DNA staining region at the 24 hr timepoint was automatically not measured (asterisks) because the non-actin staining region was 0. At least 24 wells of each condition were measured. [file 1472-6750-4-21-S1.tiff]

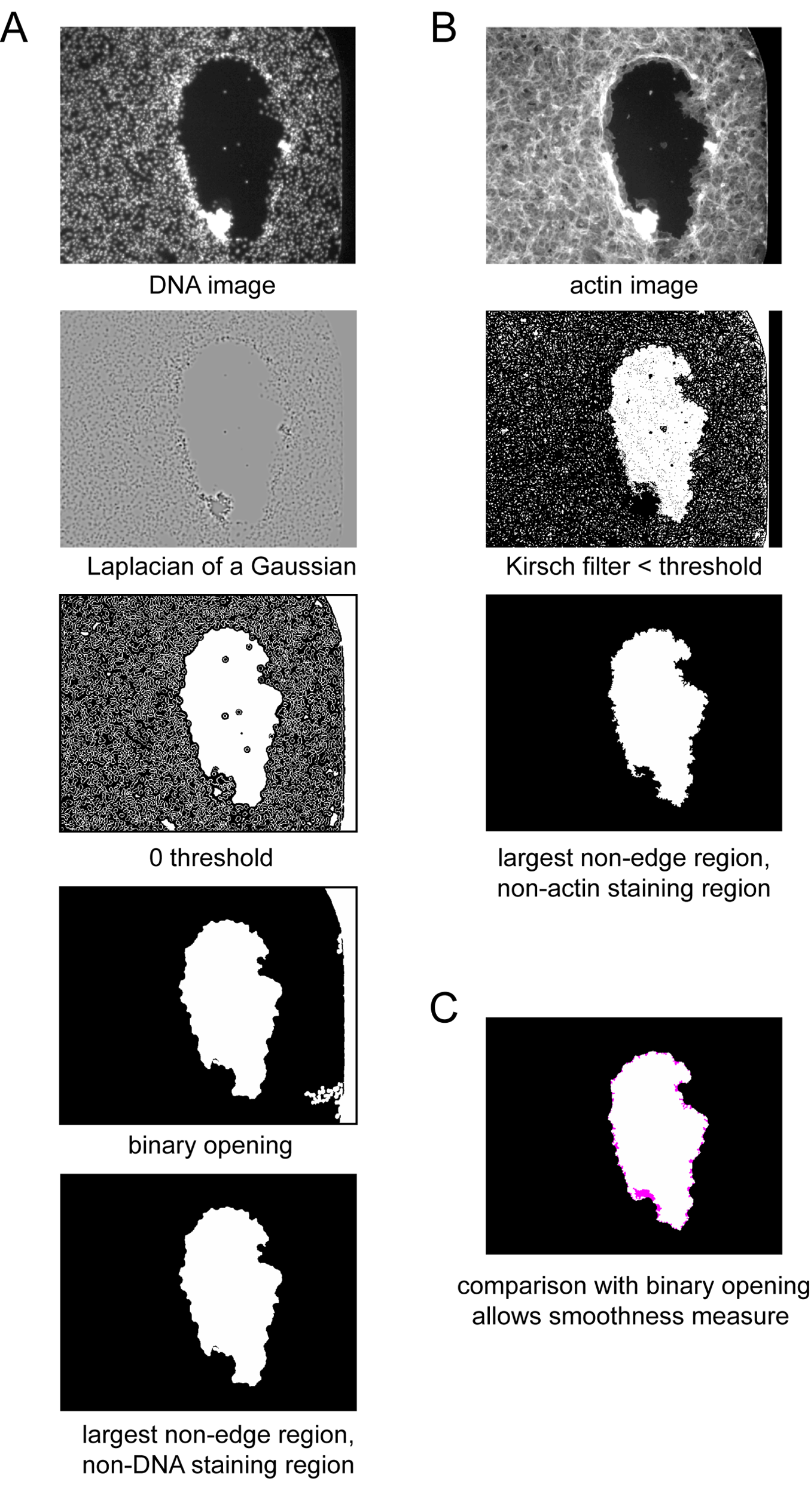

Supplement: Additional file 2 — Sample images illustrating automated analysis of wound healing images. (A) Processing of DAPI image to generate non-DNA staining region. (B) Processing of actin image to generate non-actin staining image. (C) Comparison of lamellar wound region with its morphological closing allows assessment of lamellar smoothness. [file 1472-6750-4-21-S2.tiff]
